# A Pan-Cancer Approach to Predict Responsiveness to Immune Checkpoint Inhibitors by Machine Learning

**DOI:** 10.3390/cancers11101562

**Published:** 2019-10-15

**Authors:** Maurizio Polano, Marco Chierici, Michele Dal Bo, Davide Gentilini, Federica Di Cintio, Lorena Baboci, David L. Gibbs, Cesare Furlanello, Giuseppe Toffoli

**Affiliations:** 1 Experimental and Clinical Pharmacology Unit, Centro di Riferimento Oncologico di Aviano (CRO) IRCCS, 33081 Aviano, Italy; mdalbo@cro.it (M.D.B.); federica.dicintio@cro.it (F.D.C.); lorena.baboci@cro.it (L.B.); gtoffoli@cro.it (G.T.); 2 Fondazione Bruno Kessler, 38123 Trento, Italy; chierici@fbk.eu (M.C.); furlan@fbk.eu (C.F.); 3 Department of Brain and Behavioral Sciences, University of Pavia, 27100 Pavia, Italy; davide.gentilini@unipv.it; 4 Istituto Auxologico Italiano IRCCS, Bioinformatics and Statistical Genomics Unit, 20095 Cusano Milanino, Italy; 5 Department of Life Sciences, University of Trieste, 34127 Trieste, Italy; 6 Institute for Systems Biology, 401 Terry Ave N, Seattle, WA 98109, USA; david.gibbs@systemsbiology.org

**Keywords:** immunology-pancancer, immune checkpoint inhibitor, machine learning

## Abstract

Immunotherapy by using immune checkpoint inhibitors (ICI) has dramatically improved the treatment options in various cancers, increasing survival rates for treated patients. Nevertheless, there are heterogeneous response rates to ICI among different cancer types, and even in the context of patients affected by a specific cancer. Thus, it becomes crucial to identify factors that predict the response to immunotherapeutic approaches. A comprehensive investigation of the mutational and immunological aspects of the tumor can be useful to obtain a robust prediction. By performing a pan-cancer analysis on gene expression data from the Cancer Genome Atlas (TCGA, 8055 cases and 29 cancer types), we set up and validated a machine learning approach to predict the potential for positive response to ICI. Support vector machines (SVM) and extreme gradient boosting (XGboost) models were developed with a 10×5-fold cross-validation schema on 80% of TCGA cases to predict ICI responsiveness defined by a score combining tumor mutational burden and TGF-β signaling. On the remaining 20% validation subset, our SVM model scored 0.88 accuracy and 0.27 Matthews Correlation Coefficient. The proposed machine learning approach could be useful to predict the putative response to ICI treatment by expression data of primary tumors.

## 1. Introduction

In recent years, immunotherapy has dramatically improved the treatment options in various cancers increasing the survival rates for treated patients. Among the most promising immunotherapeutic approaches there is the pharmacological manipulation of the physiologic immune checkpoints [1,2,3,4]. Immune-checkpoint blockade is the basis for the clinical antitumor activity of the most promising currently approved antibodies targeting the checkpoint molecules CTLA4 (Cytotoxic T-Lymphocyte Antigen 4) , PD1 (Programmed Cell Death 1) and PD-L1 (Programmed cell death ligand 1).Nevertheless, there are heterogeneous response rates to immune checkpoint inhibitors (ICI) [4,5,6] among the different cancer types, and also in the context of patients affected by a specific cancer. Moreover, only a minority of patients with advanced/metastatic cancer respond to ICI, thus exposing the remaining patients to potentially ineffective, toxic and costly treatments. Thus, it becomes crucial to identify predictive factors determining the response to the immunotherapeutic approaches. Intra-tumoral PD-L1 expression, evaluated by immunohistochemistry, is among the first proposed predictive biomarkers but it is not frequently successful [3,7,8]. This lack of success could be explained by the fact that a complex scenario characterized by genomic features, immune systemic state, tumor microenvironment interactions and tumor immune cell interactions is heavily involved in the efficacy of ICI [9,10,11,12,13]. Thus, it has become clear that a more robust prediction needs to involve a comprehensive investigation of the mutational and immunological aspects of the tumor diseases. Evaluation of tumor mutational burden (TMB) by whole-exome sequencing has also been proposed but it has not been demonstrated to sufficiently predict long term clinical benefits [3,4]. On the other hand, three distinct immunological phenotypes, i.e., immune inflamed, immune excluded or immune desert were proposed to categorize the majority of solid tumors in an attempt to explain their different capability to respond to ICI [8,14,15,16]. These three different immunological subtypes were associated with different transcriptomic profiles based on tumor/tumor microenvironment/immune system cell interactions. In particular: (i) immunogenomics analyses of over 10,000 tumors identified six immune subtypes, encompassing multiple cancer types, that were hypothesized to define different patterns of immune system response with predictive/prognostic relevance [17]; (ii) an immune infiltration score and a T cell infiltration score were proposed by analyzing gene expression signatures of different cancer types to define immunogenicity and potential capability to respond to ICI [18]; (iii) a tumor inflammation signature was proposed to measure pre-existing but suppressed adaptive immune response in different tumors [19]; (iv) a lack of response to ICI was associated with a signature related to transforming growth factor β (TGF-β) signaling in tumors which showed exclusion of CD8+ effector T cells from the tumor parenchyma with, on the other hand, these cells mainly located in fibroblast and collagen reach peritumoral stroma. This TGF-β signature was mainly driven by fibroblasts present in the tumor microenvironment [20]. Overall considered, this previous evidence suggested that pre-existing T cell immunity, TMB and TGF-β signaling could affect response to immunotherapy with immune checkpoint blockade. In the present study, by performing a pan-cancer analysis on gene expression data from the Cancer Genome Atlas (TCGA, 8055 cases belonging to 29 cancer types), we set up and validated a machine learning approach to predict the potential for positive response to ICI.

## 2. Results

The study included 8055 primary tumor cases for 29 cancer types from The Cancer Genome Atlas (TCGA) cohort. The number of primary tumor cases for each project is reported in Table 1.

**Figure 1:**
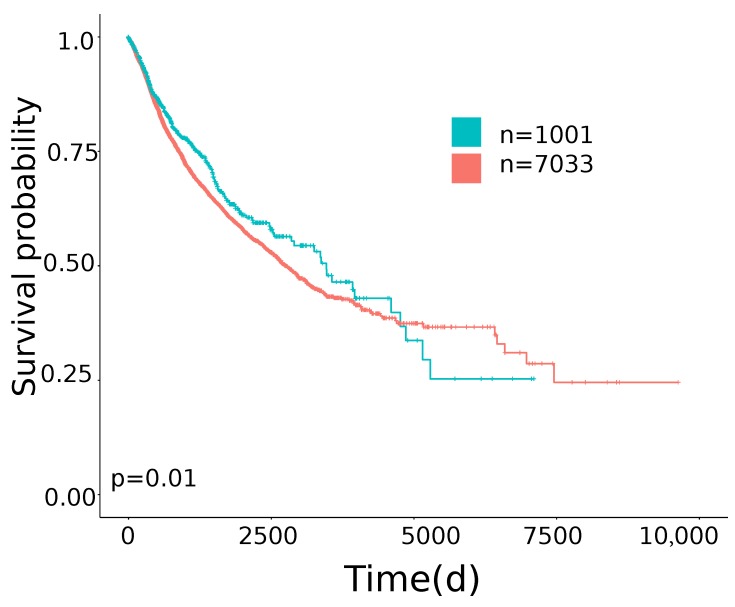
Kaplan-Meier Overall Survival (OS) curves of TMB/TGF-β score positive cases (blue line) versus TMB/TGF-β score negative cases (red line) for the 29 TCGA cancer types. Time is expressed in days; log-rank test p-value is reported.

**Figure A1:**
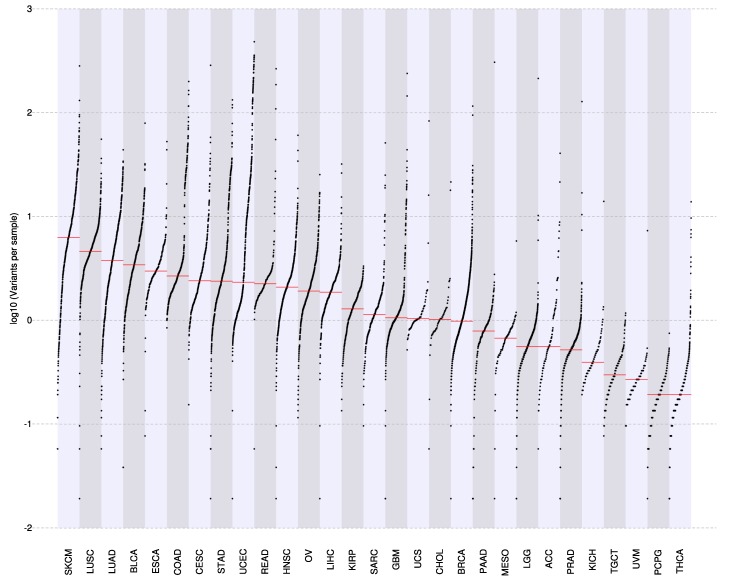
Tumor mutational burden (TMB) calculated for all the cases included in the analysis. Abbreviations of cancer cohort: adrenocortical carcinoma (ACC), bladder urothelial carcinoma (BLCA), breast invasive carcinoma (BRCA), cervical squamous cell carcinoma and endocervical adenocarcinoma (CESC), cholangiocarcinoma (CHOL), colon adenocarcinoma (COAD), esophageal carcinoma (ESCA), glioblastoma multiforme (GBM), head and neck squamous cell carcinoma (HNSC), kidney chromophobe (KICH), kidney renal papillary cell carcinoma (KIRP), brain lower grade glioma (LGG), liver hepatocellular carcinoma (LIHC), lung adenocarcinoma (LUAD), lung squamous cell carcinoma (LUSC), mesothelioma (MESO), ovarian serous cystadenocarcinoma (OV), pancreatic adenocarcinoma (PAAD), pheochromocytoma and paraganglioma (PCPG), prostate adenocarcinoma (PRAD), rectum adenocarcinoma (READ), sarcoma (SARC), skin cutaneous melanoma (SKCM), stomach adenocarcinoma (STAD), testicular germ cell tumors (TGCT), thyroid carcinoma (THCA), uterine corpus endometrial carcinoma (UCEC), uterine carcinosarcoma (UCS) and uveal melanoma (UVM).

**Figure A2:**
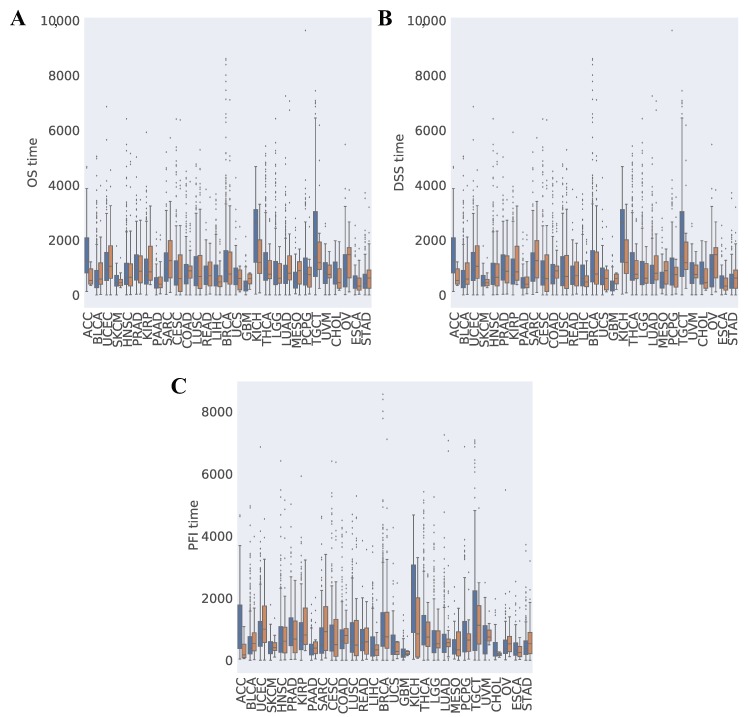
Boxplots of OS (**A**), DSS (**B**), and PFI (**C**) for TMB/TGF-β score positive cases (orange) and negative cases (blue) in the context of the different cancer types. Time is expressed in days. Abbreviations of cancer cohort: adrenocortical carcinoma (ACC), bladder urothelial carcinoma (BLCA), breast invasive carcinoma (BRCA), cervical squamous cell carcinoma and endocervical adenocarcinoma (CESC), cholangiocarcinoma (CHOL), colon adenocarcinoma (COAD), esophageal carcinoma (ESCA), glioblastoma multiforme (GBM), head and neck squamous cell carcinoma (HNSC), kidney chromophobe (KICH), kidney renal papillary cell carcinoma (KIRP), brain lower grade glioma (LGG), liver hepatocellular carcinoma (LIHC), lung adenocarcinoma (LUAD), lung squamous cell carcinoma (LUSC), mesothelioma (MESO), ovarian serous cystadenocarcinoma (OV), pancreatic adenocarcinoma (PAAD), pheochromocytoma and paraganglioma (PCPG), prostate adenocarcinoma (PRAD), rectum adenocarcinoma (READ), sarcoma (SARC), skin cutaneous melanoma (SKCM), stomach adenocarcinoma (STAD), testicular germ cell tumors (TGCT), thyroid carcinoma (THCA), uterine corpus endometrial carcinoma (UCEC), uterine carcinosarcoma (UCS) and uveal melanoma (UVM).

The distribution of TMB of the primary cases across the cancer projects are shown in Figure A1. Previous studies showed that a high TMB is associated with positive response to ICI treatments [5,8]. On the other hand, active TGF-β signaling is associated with a lack of response to ICI treatments [17,20,21]. Following this line of reasoning, we chose to classify as potentially responsive to ICI (hereafter TMB/TGF-β score positive) those cases that simultaneously had a TMB above the third quartile and the TGF-β score under the median value (TGFB_score_21050467 as described in [17]). The distribution of cases classified as responsive is reported in Table 1. Of note the tumor type with the highest number of TMB/TGF-β score positive cases was HNSC and the cancer type with the lowest number was GBM (15.57% to 4.08%). By using this TMB/TGF-β score cut off, we evaluated the overall survival (OS), disease specific survival (DSS) intervals and progression free interval (PFI) of all the cases included in the study, simultaneously considering all the TCGA projects using the last revision of the TCGA clinical data (Figure A2) [22]. Notably, as shown in Figure 1, TMB/TGF-β score positive cases showed significantly longer OS than TMB/TGF-β score negative cases (Table 2). The strongest associations were found when DSS were considered (Table 2). Moreover, TMB/TGF-β score positive cases showed significantly longer PFI (Table 2). When cases belonging to each project were considered separately different trends were observed (Table A1).

**Figure A3:**
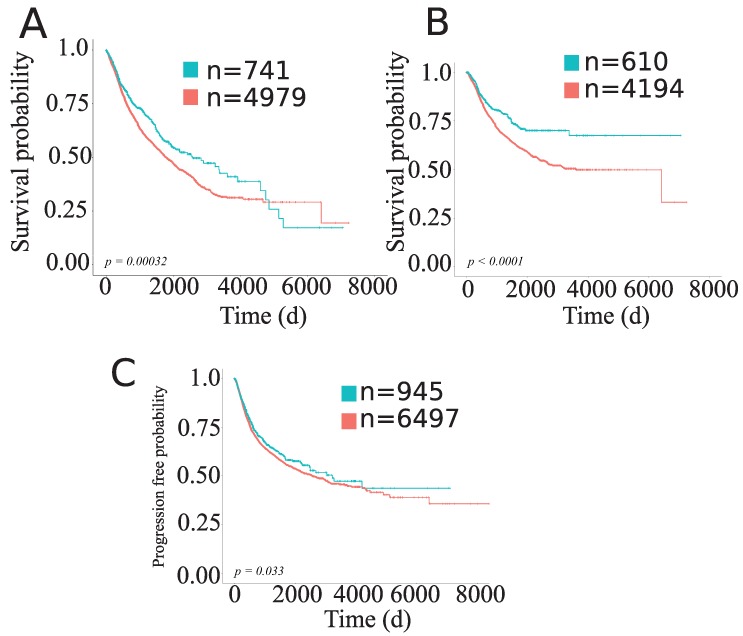
Kaplan-Meier survival curves of TMB/TGF-β score positive cases (blue line) versus TMB/TGF-β score negative cases (red line) in the context of the cancer types according to the recommendations reported in Liu et al. [22] (**A**) Overall Survival (adrenocortical carcinoma (ACC), bladder urothelial carcinoma (BLCA), uterine corpus endometrial carcinoma (UCEC), skin cutaneous melanoma (SKCM), head and neck squamous cell carcinoma (HNSC), kidney renal papillary cell carcinoma (KIRP), pancreatic adenocarcinoma (PAAD), sarcoma (SARC), cervical squamous cell carcinoma and endocervical adenocarcinoma (CESC), colon adenocarcinoma (COAD), lung squamous cell carcinoma (LUSC), liver hepatocellular carcinoma (LIHC), uterine carcinosarcoma (UCS), lung squamous cell carcinoma (LUSC), cholangiocarcinoma (CHOL), lung adenocarcinoma (LUAD), mesothelioma (MESO), uveal melanoma(UVM), ovarian serous cystadenocarcinoma (OV), esophageal carcinoma (ESCA), stomach adenocarcinoma (STAD), glioblastoma multiforme (GBM)); (**B**) Disease-specific survival (adrenocortical carcinoma (ACC), bladder urothelial carcinoma (BLCA), uterine corpus endometrial carcinoma (UCEC), skin cutaneous melanoma (SKCM), head and neck squamous cell carcinoma (HNSC), kidney renal papillary cell carcinoma (KIRP), pancreatic adenocarcinoma (PAAD), sarcoma (SARC), cervical squamous cell carcinoma and endocervical adenocarcinoma (CESC), colon adenocarcinoma (COAD), lung squamous cell carcinoma (LUSC), uterine carcinosarcoma (UCS), glioblastoma multiforme (GBM),lung adenocarcinoma (LUAD), mesothelioma (MESO), uveal melanoma (UVM), ovarian serous cystadenocarcinoma (OV), esophageal carcinoma (ESCA), stomach adenocarcinoma (STAD); (**C**) Progression-free interval (adrenocortical carcinoma (ACC), bladder urothelial carcinoma (BLCA), uterine corpus endometrial carcinoma (UCEC), skin cutaneous melanoma (SKCM), head and neck squamous cell carcinoma (HNSC), prostate adenocarcinoma (PRAD), pancreatic adenocarcinoma (PAAD), kidney renal papillary cell carcinoma (KIRP), sarcoma (SARC), cervical squamous cell carcinoma and endocervical adenocarcinoma (CESC), colon adenocarcinoma (COAD), lung squamous cell carcinoma (LUSC), liver hepatocellular carcinoma (LIHC), breast invasive carcinoma (BRCA), thyroid carcinoma (THCA), uterine carcinosarcoma (UCS), brain lower grade glioma (LGG), cholangiocarcinoma (CHOL), lung adenocarcinoma (LUAD), testicular germ cell tumors (TGCT), mesothelioma (MESO), ovarian serous cystadenocarcinoma (OV), esophageal carcinoma (ESCA), stomach adenocarcinoma (STAD)). Time is expressed in days; log-rank test *p*-value is reported.

Liu et al. [22] presented a curated and filtered analysis for clinical and survival outcome data defining the assessment and recommended use of the endpoints. Noteworthy, TMB/TGF-β score positive cases showed significantly longer OS, DSS and PFI than TMB/TGF-β score negative cases when using a restricted subgroup from 29 cancer types as recommended by Liu et al. [22] (Figure A3A–C).

To evaluate the immune-related features of gene expression signatures of TMB/TGF-β score positive cases, we classified the cases included in the study according to the six immune subtypes defined in Thorsson et al. [17], where a multi-omic analysis of TCGA datasets allowed the definition of subtypes ( C1 (wound healing), C2 (IFN-γ dominant), C3 (inflammatory), C4 (lymphocyte depleted), C5 (immunologically quiet), C6 (TGF-β dominant) ) useful to classify cancer cases across different cancer types according to distinct immune signatures.

**Figure 2:**
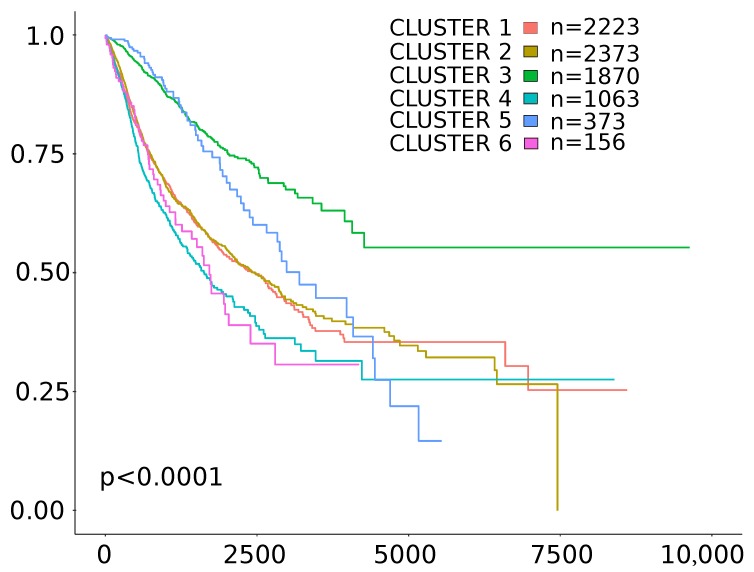
Kaplan-Meier Overall Survival (OS) curves of the six immune subtypes described in Thorsson et al. [17] using the case cohort included in the present study. Time is expressed in days; log-rank test p-value is reported.

**Figure A4:**
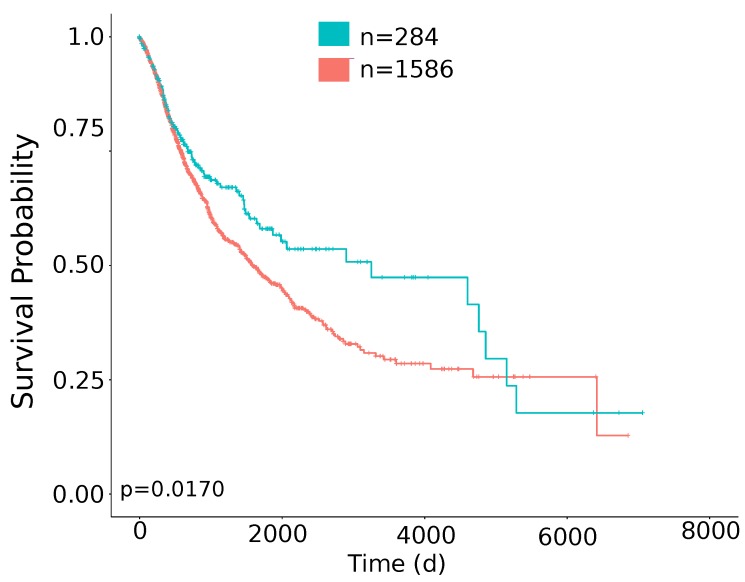
Kaplan-Meier Overall Survival (OS) curves of TMB/TGF-β score positive cases (blue line) versus TMB/TGF-β score negative cases (red line) belonging to the Cluster 2, in the context of the 20 TCGA cancer types according to the recommendations reported in Liu et al. [22] ( adrenocortical carcinoma (ACC), bladder urothelial carcinoma (BLCA), cervical squamous cell carcinoma and endocervical adenocarcinoma (CESC), cholangiocarcinoma (CHOL), colon adenocarcinoma (COAD), esophageal carcinoma (ESCA), head and neck squamous cell carcinoma (HNSC),kidney renal papillary cell carcinoma (KIRP),kidney chromophobe (KICH), liver hepatocellular carcinoma (LIHC), lung adenocarcinoma (LUAD), lung squamous cell carcinoma (LUSC), mesothelioma (MESO), ovarian serous cystadenocarcinoma (OV), pancreatic adenocarcinoma (PAAD), sarcoma (SARC), skin cutaneous melanoma (SKCM), stomach adenocarcinoma (STAD), uterine corpus endometrial carcinoma (UCEC), uterine carcinosarcoma (UCS)). Time is expressed in days; log-rank test *p*-value is reported.

To perform this classification we used an implemented version of the tool proposed in [23]. The number of cases found in each subtype by performing this analysis is reported in Table A2. TMB/TGF-β score positive cases were found enriched in the C2 subtype (IFN${}_{\gamma }$ dominant) characterized by highly mutated tumors. Moreover, while constructing our classification score, we observed a very low number of cases of TMB/TGF-β score positive cases in the C6 (TGF-β dominant) subtype (Table A2) [17]. By considering the entire TCGA cohort, clinical outcomes were in line with those reported in [17] (Figure 2). Notably, within both the favorable prognosis group Cluster 2 and the unfavorable prognosis group Cluster 4, TMB/TGF-β score positive cases showed significantly longer OS intervals than the TMB/TGF-β score negative counterparts (Table 3). Moreover, in Cluster 2, again TMB/TGF-β score positive cases showed significantly longer OS intervals than the TMB/TGF-β score negative counterparts by considering only the subgroup of 20 cancer types according to the recommendations reported in [22] (Figure A4).

**Figure A5:**
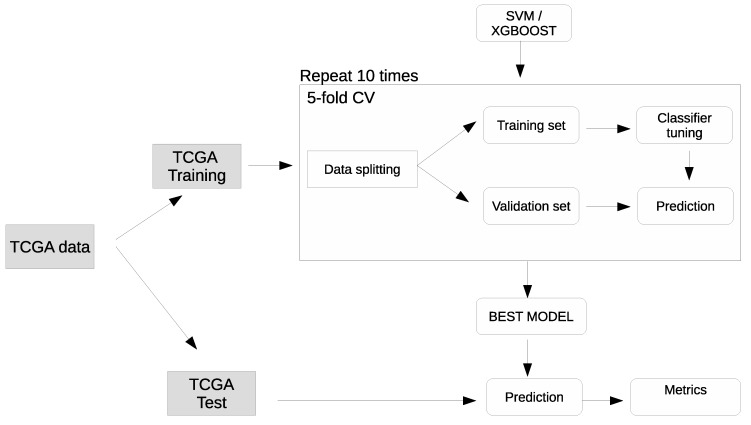
Graphical representation of the pipeline used to develop the model for classifying the TMB/TGF-β score positive cases.

To select the optimal classification model, two machine learning algorithms were used: Support Vector Machines (SVM) and optimized distributed gradient boosting (XGboost). Following the approach depicted in Figure A5, the TCGA transcriptomics data was split into training and test sets. The training set was used for model development, within a 10×5fold Stratified Cross Validation [24], and the test set was used for assessing the model performance. As evaluation metrics, accuracy (ACC) and the Matthews correlation coefficient (MCC) [25,26] were used.

**Figure A6:**
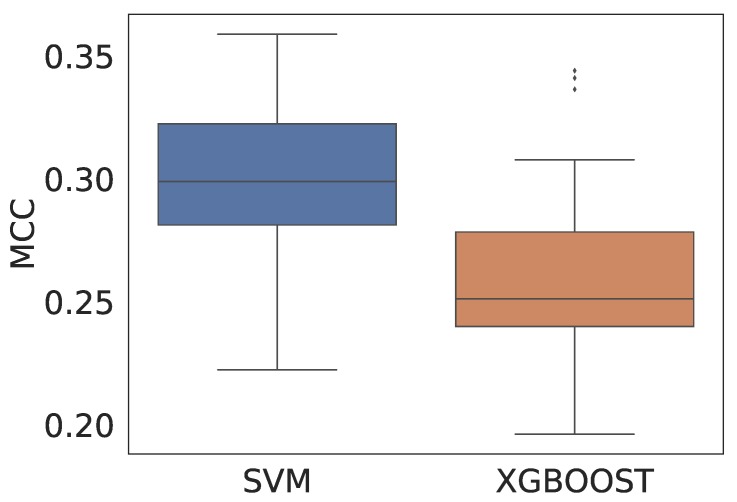
Boxplots of cross-validation MCC values for SVM and XGBoost models. *p* = 0.00103, Kruskal-Wallis multiple test.

The classifiers were trained using the genes extracted in [17] (2387 genes grouped in 160 signatures). The SVM model achieved a mean cross-validation MCC of 0.296 (95% boostrapped confidence interval: 0.287-0.306), significantly higher than the XGBoost model with a mean cross-validation MCC of 0.260 (0.250-0.269) (Kruskal-Wallis *p* = 0.001; Table 4, Figure A6). On the test set, the SVM model achieved MCC=0.271 (Table 4).

## 3. Discussion

The use of ICI has changed the clinical management of tumor-affected patients, although heterogeneous response rates have been found for treated patients across different cancer types as well as for patients affected by a specific tumor type. In particular, ICI might also improve the treatment of urothelial cancer, gastric cancer, colorectal cancer, lung cancer and breast cancer considering the promising results achieved so far and the relatively low efficacy of currently available treatments [27,28,29,30,31]. Given this heterogeneous response, there is the clinical need for predictive biomarkers for the definition of responsiveness to ICI treatments. Currently employed biomarkers, such as PD-L1 expression levels and TMB, have shown an incomplete predictive performance [4]. An alternative point of view could be represented by the introduction of complex biomarkers simultaneously evaluating multiple tumor/tumor microenvironment/immune system features [12,13].

To this aim, starting from genomic, transcriptomic and proteomic data, machine learning approaches could be useful to obtain accurate prediction models for response to ICI treatments [21]. In particular, different approaches, sub-typing oriented and based mainly on gene expression patterns, have been recently proposed [18,21,32,33,34]. In these studies, machine learning supervised algorithms have been generally trained to match a known phenotype (for example, established by microscopy or with clinical features) to genetic patterns. In the last years, comprehensive immunogenomic analyses of different cancer types, based on TCGA data, have been proposed to characterize tumor heterogeneity in terms of immune-related features, possibly influencing the capability to respond to ICI treatments [17].

Different studies suggested that TMB is associated with survival prognosis in many cancer types, given the association with the formation of neoantigens capable of stimulating anti-cancer T lymphocyte clones. Nevertheless, the mechanism underlying this association could lie in the marked differences in immune cell infiltration densities and immune activities depending on tumor microenvironment immunosuppressive cell populations, T cell exhaustion and tumor associated stromal tissue [5,19,33,34,35]. Another important point for the different behaviors according to TMB reported in literature is that the method to calculate TMB is not univocal [36,37]. A combination of 2 biomarkers, one dependent from the tumor intrinsic mutational state and one related to the tumor microenvironment, could therefore identify patients that can potentially benefit from ICI. To this aim, to perform the pan-cancer analysis we chose to use a surrogate (i.e., TMB and TGF-β score) to define cases putatively responsive to ICI treatments. The choice to use this surrogate is due to the fact that the comprehensive TCGA case cohort is not homogeneous in terms of employed anti-cancer treatments, with only a minority of cases undergoing ICI treatments.

Thus, to derive a label to be used by a machine learning classifier, we defined as potentially responsive to ICI those cases that simultaneously had a TMB above the third quartile and the TGF-β score under the median value (TGFB_score_21050467 as described in [17]). The choice to use this phenotype to classify cases putatively responsive to ICI could be considered in keeping with the fact that when primary cases of all the 29 TCGA cancer types were simultaneously considered, TMB/TGF-β- score positive cases showed significantly longer OS, DSS and PFI intervals than TMB/TGF-β score negative cases, irrespective of the type of cancer, the clinical and molecular features and the treatment managements of the analyzed cases (Figure 1). We developed a classification model using as predictors the 2387 genes associated with 160 immuno-related signatures reported in Thorsson et al. [17].

To evaluate classification of TMB/TGF-β score positive cases, we compared SVM and XGBoost algorithms. The best classification performance was obtained using SVM. These results could be explained by the fact that SVM is usually robust, even when the training sample cohort has some bias. The obtained MCC prompts to suggest a mild correlation of the TMB/TGF-β score used to identify responsiveness to ICI with the features used to create the model. Previous proposed methods used different algorithms and combinations of data obtained from different databases [18,21,32,33,34]. In this context, we focused only on primary tumors and transcriptomics data choosing two surrogates of possible response to ICI. A limitation of our proposed method could be represented by the high number of genes used to classify the putative responsiveness to ICI. However, similar approaches using high number of genes or multi-omic combinations with high numbers of data have been previously published [17,18,21,32,33,34]. On the other hand, comparison among our proposed model and previously published models seems to be not feasible given the different starting data and different employed approaches. Nevertheless, the proposed model could be naturally extended with multi-modal inputs by adding appropriate embeddings, in particular clinical variables and image data. On the other hand, it is noteworthy that the proposed machine learning classifier could be useful to stratify patients according to the putative responsiveness to ICI treatment, also considering cancer patients comprehensively characterized by immune-related features associated with a favorable prognosis such as those belonging to immune subtype C2.

## 4. Materials and Methods

### 4.1. Datasets

The Cancer Genome Atlas (TCGA) RNA sequencing (RNA-seq) count data (FPKM-UQ) was downloaded (February 2019) from the GDC data portal (portal.gdc.cancer.gov) using the *GenomicDataCommons* Bioconductor package [38]. We downloaded RNA-Seq data of 29 primary tumors described by Table 1. In the following list all the abbreviations of the cancer cohorts used in this study are reported: adrenocortical carcinoma (ACC), bladder urothelial carcinoma (BLCA), breast invasive carcinoma (BRCA), cervical squamous cell carcinoma and endocervical adenocarcinoma (CESC), cholangiocarcinoma (CHOL), colon adenocarcinoma (COAD), esophageal carcinoma (ESCA), glioblastoma multiforme (GBM), head and neck squamous cell carcinoma (HNSC), kidney chromophobe (KICH), kidney renal papillary cell carcinoma (KIRP), brain lower grade glioma (LGG), liver hepatocellular carcinoma (LIHC), lung adenocarcinoma (LUAD), lung squamous cell carcinoma (LUSC), mesothelioma (MESO), ovarian serous cystadenocarcinoma (OV), pancreatic adenocarcinoma (PAAD), pheochromocytoma and paraganglioma (PCPG), prostate adenocarcinoma (PRAD), rectum adenocarcinoma (READ), sarcoma (SARC), skin cutaneous melanoma (SKCM), stomach adenocarcinoma (STAD), testicular germ cell tumors (TGCT), thyroid carcinoma (THCA), uterine corpus endometrial carcinoma (UCEC), uterine carcinosarcoma (UCS) and uveal melanoma(UVM).

The tumor mutational burden (TMB) was calculated from the MC3 Public MAF [39] file as described by Alexandrov and colleagues [36,40]. To characterize intratumoral immune states, we scored the 160 immune expression signatures as described by Thorsson and colleagues [17]. We used the signature published on the “Immune-Subtype-Clustering” GitHub repository [41] and then we tested the improved version of the tool [23].

For each cancer cohort, cases were labeled as responsive if they simultaneously had TMB above the third quartile and TGF-β score under the median value (TGFB_score_21050467 as decribed in Thorsson et al. [17]).

### 4.2. Machine learning methods

For the selection of an initial classification model, we evaluated the performance of two supervised learning methods, namely support vector machines (SVM) and extreme gradient boosting (XGBoost). The optimal hyperparameters were selected with a grid search across a space of model-specific parameters. The data were split beforehand into 80% training and 20% test partitions. All models were developed in a 10× 5-fold cross validation (CV) schema on the training partition using the 2387 genes reported by Thorsson et al [17]. Performance was assessed in terms of accuracy (ACC) and Matthews Correlation Coefficient (MCC) [25,26], the performance metric that effectively summarizes in a single value the confusion matrix of a classification task, even when the classes are imbalanced. MCC values are in the [−1,1] range, where 1 means perfect classification, −1 perfect misclassification, and 0 random prediction or classification of every sample to the largest class. The overall performance in cross-validation is evaluated across all CV iterations as average MCC and ACC with 95% Studentized bootstrap confidence intervals (CI), and on the test partition as MCC and ACC. The classification pipeline was also run with randomized labels as a sanity check for unwanted selection bias effects: in a procedure unaffected by systematic bias, the average MCC should be close to 0. Data were log2-transformed and standardized to zero mean and unit variance before classification; in order to avoid potential information leakage, the standardization parameters from the training set were used for rescaling both training and test subsets.

### 4.3. Computational Details

The classification pipeline was built on top of the Scikit Learn library 0.20.3 [42] using Python 3.6. All the experiments were run on a 32-core Intel Core i7 workstation with 128GB of RAM running CentOS 7.5. Cox regression and Kaplan-Meier survival curves were computed using R (version 3.6.1 ) with *survival* and *survminer* packages. Survival curves were compared with the log-rank test. Survival analysis were performed in cases for which all census data were available according to Liu et al. [22]

## 5. Conclusions

Balancing between immunostimulative and immunosuppressive factors exerting a role in the tumor/tumor microenvironment/immune system crosstalk can influence the capability to respond to ICI treatment of cancer-affected patients. This results in heterogeneous response rates among different cancer types but also in the context of a specific cancer. In this complex scenario, there is the need to efficiently predict the capability of patients to respond to these immunotherapeutic approaches. Here, we proposed a machine learning approach to comprehensively investigate mutational and immunological aspects of tumor diseases. This could be useful to efficiently predict the putative response to ICI treatment by expression data of primary tumors.

## Figures and Tables

**Table 1:** Cases included in the study from The Cancer Genome Atlas (TCGA) cohorts for 29 cancer types and frequency of TMB/TGF-β score positive cases in the context of each cancer type.

Cohort	Cancer Type Full Name	Number of Cases	Percentage of TMB/TGF-β Score Positive Cases
HNSC	head and neck squamous cell carcinoma	488	15.57
LUSC	lung squamous cell carcinoma	476	14.71
LIHC	liver hepatocellular carcinoma	350	14.29
UCEC	uterine corpus endometrial carcinoma	511	14.29
CESC	cervical squamous cell carcinoma and endocervical adenocarcinoma	282	14.18
BLCA	bladder urothelial carcinoma	397	14.11
STAD	stomach adenocarcinoma	349	13.75
PRAD	prostate adenocarcinoma	401	13.72
KIRP	kidney renal papillary cell carcinoma	267	13.48
BRCA	breast invasive carcinoma	970	13.30
ESCA	esophageal carcinoma	151	13.25
MESO	mesothelioma	77	12.99
SKCM	skin cutaneous melanoma	103	12.62
UCS	uterine carcinosarcoma	56	12.50
UVM	uveal melanoma	80	12.50
READ	rectum adenocarcinoma	126	11.90
THCA	thyroid carcinoma	481	11.85
COAD	colon adenocarcinoma	383	11.75
PAAD	pancreatic adenocarcinoma	146	11.64
CHOL	cholangiocarcinoma	35	11.43
TGCT	testicular germ cell tumors	143	11.19
PCPG	pheochromocytoma and paraganglioma	177	10.73
LUAD	lung adenocarcinoma	450	10.22
SARC	sarcoma	201	9.95
KICH	kidney chromophobe	64	9.38
LGG	brain lower grade glioma	501	7.98
OV	ovarian serous cystadenocarcinoma	165	7.88
ACC	adrenocortical carcinoma	78	7.69
GBM	glioblastoma multiforme	147	4.08

**Table 2:** Univariate Cox regression analysis of OS, DSS, PFI in the entire cohort included in the study.

Endpoint	Status	Number of Samples	HR	95% CI for HR	*p* Value
OS	TMB/TGF-β score positive	*n* = 8007	0.86	0.75–0.98	0.01
DSS	TMB/TGF-β score positive	*n* = 7741	0.79	0.67–0.93	0.0056
PFI	TMB/TGF-β score positive	*n* = 8007	0.89	0.79–0.99	0.059

Abbreviations: OS, overall survival; DSS, disease specific survival; PFI, progression free survival; HR, hazard ratio; CI, confidence interval.

**Table 3:** Univariate Cox regression analysis of OS on the six immune subtype clusters described in Thorsson et al. [17]

Cluster	Status	Number of Samples	HR	95% CI for HR	*p* Value
**Cluster 1**	TMB/TGF-β score positive	*n* = 2200	0.82	0.64–1	0.11
**Cluster 2**	TMB/TGF-β score positive	*n* = 2357	0.76	0.61–0.93	0.0095
**Cluster 3**	TMB/TGF-β score positive	*n* = 1867	0.84	0.53–1.3	0.48
**Cluster 4**	TMB/TGF-β score positive	*n* = 1061	0.72	0.52–0.99	0.044
**Cluster 5**	TMB/TGF-β score positive	*n* = 368	1.7	0.71–3.9	0.24
**Cluster 6**	TMB/TGF-β score positive	*n* = 154	2.7	1.1–6.8	0.037

Abbreviations: OS, overall survival; HR, hazard ratio; CI, confidence interval.

**Table 4:** Model metrics in cross-validation (mean with confidence intervals) and on the test set. ACC: accuracy; MCC: Matthews Correlation Coefficient; CI: 95% studentized bootstrap confidence interval.

Model	ACC (CI)	ACC Test	MCC (CI)	MCC Test
**SVM**	0.879 (0.878–0.881)	0.877	0.296 (0.287–0306)	0.271
**XGBoost**	0.878 (0.877–0.880)	0.879	0.260 (0.250–0.269)	0.260

## Abbreviations

The following abbreviations are used in this manuscript:

ICI	immune checkpoint inhibitors
TCGA	The Cancer Genome Atlas
XGboost	Extreme distributed gradient boosting library
SVM	Support Vector Machine
TMB	Tumor Mutational Burden
CI	confidence intervals
HR	Hazard ratio

## Appendix A

**Table A1:** Univariate Cox proportional hazards model analysis of survival for each cancer type included in the study.

Cancer Types	Label	Number of Samples	HR	95% CI for HR	*p* Value
**UVM**	TMB/TGF-β score positive	*n* = 80	0.3	0.04–2.2	0.24
**SKCM**	TMB/TGF-β score positive	*n* = 103	0.45	0.11–2	0.29
**GBM**	TMB/TGF-β score positive	*n* = 146	0.69	0.28–1.7	0.42
**LIHC**	TMB/TGF-β score positive	*n* = 349	1	0.59–1.8	0.91
**SARC**	TMB/TGF-β score positive	*n* = 201	0.85	0.41–1.8	0.66
**PCPG**	TMB/TGF-β score positive	*n* = 177	4.9	0.8–30	0.085
**TCGT**	TMB/TGF-β score positive	*n* = 127	1.5	0.89–2.6	0.12
**THCA**	TMB/TGF-β score positive	*n* = 481	0.87	0.11–6.7	0.89
**PAAD**	TMB/TGF-β score positive	*n* = 146	1	0.53–1.9	0.96
**PRAD**	TMB/TGF-β score positive	*n* = 410	3	0.74–12	0.12
**UCEC**	TMB/TGF-β score positive	*n* = 542	0.25	0.092–0.69	0.007
**CHOL**	TMB/TGF-β score positive	*n* = 35	2.2	0.69–7	0.18
**KICH**	TMB/TGF-β score positive	*n* = 64	6.4	1.6–26	0.0091
**BLCA**	TMB/TGF-β score positive	*n* = 412	0.67	0.42–1.1	0.096
**KIRP**	TMB/TGF-β score positive	*n* = 266	0.27	0.064–1.1	0.071
**HNSC**	TMB/TGF-β score positive	*n* = 488	1.1	0.8–1.6	0.48
**CESC**	TMB/TGF-β score positive	*n* = 282	0.48	0.19–1.2	0.11
**BRCA**	TMB/TGF-β score positive	*n* = 1009	0.94	0.57–1.6	0.81
**OV**	TMB/TGF-β score positive	*n* = 164	0.67	0.31–1.4	0.31
**LGG**	TMB/TGF-β score positive	*n* = 499	0.87	0.38–2	0.74
**LUAD**	TMB/TGF-β score positive	*n* = 492	0.58	0.33–1	0.05
**ESCA**	TMB/TGF-β score positive	*n* = 151	1.3	0.65–2.7	0.45
**READ**	TMB/TGF-β score positive	*n* = 125	0.86	0.19–3.9	0.85
**LUSC**	TMB/TGF-β score positive	*n* = 484	0.79	0.52–1.2	0.28
**COAD**	TMB/TGF-β score positive	*n* = 462	0.73	0.39–1.4	0.33
**UCS**	TMB/TGF-β score positive	*n* = 56	1.1	0.4–3.3	0.8
**MESO**	TMB/TGF-β score positive	*n* = 76	0.5	0.21–1.2	0.11
**ACC**	TMB/TGF-β score positive	*n* = 78	6.4	2.3–18	4 $\times 10^{-4}$
**STAD**	TMB/TGF-β score positive	*n* = 345	0.62	0.35–1.1	0.088

Abbreviations of cancer cohort: adrenocortical carcinoma (ACC), bladder urothelial carcinoma (BLCA), breast invasive carcinoma (BRCA), cervical squamous cell carcinoma and endocervical adenocarcinoma (CESC), cholangiocarcinoma (CHOL), colon adenocarcinoma (COAD), esophageal carcinoma (ESCA), glioblastoma multiforme (GBM), head and neck squamous cell carcinoma (HNSC), kidney chromophobe (KICH), kidney renal papillary cell carcinoma (KIRP), brain lower grade glioma (LGG), liver hepatocellular carcinoma (LIHC), lung adenocarcinoma (LUAD), lung squamous cell carcinoma (LUSC), mesothelioma (MESO), ovarian serous cystadenocarcinoma (OV), pancreatic adenocarcinoma (PAAD), pheochromocytoma and paraganglioma (PCPG), prostate adenocarcinoma (PRAD), rectum adenocarcinoma (READ), sarcoma (SARC), skin cutaneous melanoma (SKCM), stomach adenocarcinoma (STAD), testicular germ cell tumors (TGCT), thyroid carcinoma (THCA), uterine corpus endometrial carcinoma (UCEC), uterine carcinosarcoma (UCS) and uveal melanoma (UVM).

**Table A2:** Distribution of TMB/TGF-β score positive and negative cases in each Cluster subtype as described by Thorsson et al. [17] modify table.

Cluster Subtype	TMB/TGF-β Score Negative	TMB/TGF-β Score Positive	Total
1	1957	266	2223
2	1994	379	2373
3	1704	166	1870
4	913	150	1063
5	334	36	370
6	149	7	156

## References

[1] Friedrich M., Jasinski-Bergner S., Lazaridou M.F., Subbarayan K., Massa C., Tretbar S., Mueller A., Handke D., Biehl K., Bukur J. (2019). Tumor-induced escape mechanisms and their association with resistance to checkpoint inhibitor therapy. Cancer Immunol. Immunother..

[2] Costantini A., Takam Kamga P., Dumenil C., Chinet T., Emile J.F., Giroux Leprieur E. (2019). Plasma Biomarkers and Immune Checkpoint Inhibitors in Non-Small Cell Lung Cancer: New Tools for Better Patient Selection?. Cancers.

[3] Havel J.J., Chowell D., Chan T.A. (2019). The evolving landscape of biomarkers for checkpoint inhibitor immunotherapy. Nat. Rev. Cancer.

[4] Darvin P., Toor S.M., Sasidharan Nair V., Elkord E. (2018). Immune checkpoint inhibitors: Recent progress and potential biomarkers. Exp. Mol. Med..

[5] Wang X., Li M. (2019). Correlate tumor mutation burden with immune signatures in human cancers. BMC Immunol..

[6] Prat A., Navarro A., Paré L., Reguart N., Galván P., Pascual T., Martínez A., Nuciforo P., Comerma L., Alos L. (2017). Immune-Related Gene Expression Profiling After PD-1 Blockade in Non-Small Cell Lung Carcinoma, Head and Neck Squamous Cell Carcinoma, and Melanoma. Cancer Res..

[7] Zhang L., Jones-O’Connor M., Awadalla M., Zlotoff D.A., Thavendiranathan P., Groarke J.D., Villani A.C., Lyon A.R., Neilan T.G. (2019). Cardiotoxicity of Immune Checkpoint Inhibitors. Curr. Treat. Options Cardiovasc. Med..

[8] Maleki Vareki S. (2018). High and low mutational burden tumors versus immunologically hot and cold tumors and response to immune checkpoint inhibitors. J. Immunother. Cancer.

[9] Buchbinder E.I., Desai A. (2016). CTLA-4 and PD-1 Pathways: Similarities, Differences, and Implications of Their Inhibition. Am. J. Clin. Oncol..

[10] Lawrence M.S., Stojanov P., Polak P., Kryukov G.V., Cibulskis K., Sivachenko A., Carter S.L., Stewart C., Mermel C.H., Roberts S.A. (2013). Mutational heterogeneity in cancer and the search for new cancer-associated genes. Nature.

[11] Li B., Li T., Pignon J.C., Wang B., Wang J., Shukla S.A., Dou R., Chen Q., Hodi F.S., Choueiri T.K. (2016). Landscape of tumor-infiltrating T cell repertoire of human cancers. Nat. Genet..

[12] Schulz M., Salamero-Boix A., Niesel K., Alekseeva T., Sevenich L. (2019). Microenvironmental Regulation of Tumor Progression and Therapeutic Response in Brain Metastasis. Front. Immunol..

[13] Chen D.S., Mellman I. (2017). Elements of cancer immunity and the cancer-immune set point. Nature.

[14] Sharma P., Hu-Lieskovan S., Wargo J.A., Ribas A. (2017). Primary, Adaptive, and Acquired Resistance to Cancer Immunotherapy. Cell.

[15] Khong H.T., Restifo N.P. (2002). Natural selection of tumor variants in the generation of “tumor escape” phenotypes. Nat. Immunol..

[16] Kather J.N., Suarez-Carmona M., Charoentong P., Weis C.A., Hirsch D., Bankhead P., Horning M., Ferber D., Kel I., Herpel E. (2018). Topography of cancer-associated immune cells in human solid tumors. eLife.

[17] Thorsson V., Gibbs D.L., Brown S.D., Wolf D., Bortone D.S., Yang T.H.O., Porta-Pardo E., Gao G., Plaisier C.L., Eddy J.A. (2018). The Immune Landscape of Cancer. Immunity.

[18] Şenbabaoğlu Y., Gejman R.S., Winer A.G., Liu M., Van Allen E.M., de Velasco G., Miao D., Ostrovnaya I., Drill E., Luna A. (2016). Tumor immune microenvironment characterization in clear cell renal cell carcinoma identifies prognostic and immunotherapeutically relevant messenger RNA signatures. Genome Biol..

[19] Danaher P., Warren S., Lu R., Samayoa J., Sullivan A., Pekker I., Wallden B., Marincola F.M., Cesano A. (2018). Pan-cancer adaptive immune resistance as defined by the Tumor Inflammation Signature (TIS): Results from The Cancer Genome Atlas (TCGA). J. Immunother. Cancer.

[20] Mariathasan S., Turley S.J., Nickles D., Castiglioni A., Yuen K., Wang Y., Kadel E.E., Koeppen H., Astarita J.L., Cubas R. (2018). TGFB attenuates tumour response to PD-L1 blockade by contributing to exclusion of T cells. Nature.

[21] Charoentong P., Finotello F., Angelova M., Mayer C., Efremova M., Rieder D., Hackl H., Trajanoski Z. (2017). Pan-cancer Immunogenomic Analyses Reveal Genotype-Immunophenotype Relationships and Predictors of Response to Checkpoint Blockade. Cell Rep..

[22] Liu J., Lichtenberg T., Hoadley K.A., Poisson L.M., Lazar A.J., Cherniack A.D., Kovatich A.J., Benz C.C., Levine D.A., Lee A.V. (2018). An Integrated TCGA Pan-Cancer Clinical Data Resource to Drive High-Quality Survival Outcome Analytics. Cell.

[23] Gibbs D.L. (2019). An R package for classification of immune subtypes, in cancer, using gene expression data.

[24] Hastie T., Tibshirani R., Friedman J. (2001). The Elements of Statistical Learning: Data Mining, Inference, and Prediction.

[25] Matthews B.W. (1975). Comparison of the predicted and observed secondary structure of T4 phage lysozyme. Biochim. Biophys. Acta.

[26] Jurman G., Riccadonna S., Furlanello C. (2012). A comparison of MCC and CEN error measures in multi-class prediction. PLoS ONE.

[27] Bonotto M., Garattini S.K., Basile D., Ongaro E., Fanotto V., Cattaneo M., Cortiula F., Iacono D., Cardellino G.G., Pella N. (2017). Immunotherapy for gastric cancers: Emerging role and future perspectives. Expert Rev. Clin. Pharmacol..

[28] Visconti R., Morra F., Guggino G., Celetti A. (2017). The between Now and Then of Lung Cancer Chemotherapy and Immunotherapy. Int. J. Mol. Sci..

[29] Emens L.A. (2018). Breast Cancer Immunotherapy: Facts and Hopes. Clin. Cancer Res..

[30] Basile D., Garattini S.K., Bonotto M., Ongaro E., Casagrande M., Cattaneo M., Fanotto V., De Carlo E., Loupakis F., Urbano F. (2017). Immunotherapy for colorectal cancer: Where are we heading?. Expert Opin. Biol. Ther..

[31] Cattrini C., Dellepiane C., Cavo A., Buzzatti G., Tolomeo F., Messina C., Boccardo F. (2016). Immunotherapy for genitourinary cancer: State of the art and new perspectives. Anticancer Drugs.

[32] Angelova M., Charoentong P., Hackl H., Fischer M.L., Snajder R., Krogsdam A.M., Waldner M.J., Bindea G., Mlecnik B., Galon J. (2015). Characterization of the immunophenotypes and antigenomes of colorectal cancers reveals distinct tumor escape mechanisms and novel targets for immunotherapy. Genome Biol..

[33] Tamborero D., Rubio-Perez C., Muiños F., Sabarinathan R., Piulats J.M., Muntasell A., Dienstmann R., Lopez-Bigas N., Gonzalez-Perez A. (2018). A Pan-cancer Landscape of Interactions between Solid Tumors and Infiltrating Immune Cell Populations. Clin. Cancer Res..

[34] McGranahan N., Furness A.J.S., Rosenthal R., Ramskov S., Lyngaa R., Saini S.K., Jamal-Hanjani M., Wilson G.A., Birkbak N.J., Hiley C.T. (2016). Clonal neoantigens elicit T cell immunoreactivity and sensitivity to immune checkpoint blockade. Science.

[35] Ma W., Gilligan B.M., Yuan J., Li T. (2016). Current status and perspectives in translational biomarker research for PD-1/PD-L1 immune checkpoint blockade therapy. J. Hematol. Oncol..

[36] Meléndez B., Van Campenhout C., Rorive S., Remmelink M., Salmon I., D’Haene N. (2018). Methods of measurement for tumor mutational burden in tumor tissue. Transl. Lung Cancer Res..

[37] Chalmers Z.R., Connelly C.F., Fabrizio D., Gay L., Ali S.M., Ennis R., Schrock A., Campbell B., Shlien A., Chmielecki J. (2017). Analysis of 100,000 human cancer genomes reveals the landscape of tumor mutational burden. Genome Med..

[38] Martin T.M., Davis S.R. GenomicDataCommons R-Package | NCI Genomic Data Commons Access 2019. https://bioconductor.org/packages/GenomicDataCommons,http://github.com/Bioconductor/GenomicDataCommons.

[39] Ellrott K., Bailey M.H., Saksena G., Covington K.R., Kandoth C., Stewart C., Hess J., Ma S., Chiotti K.E., McLellan M. (2018). Scalable Open Science Approach for Mutation Calling of Tumor Exomes Using Multiple Genomic Pipelines. Cell Syst..

[40] Alexandrov L.B., Nik-Zainal S., Wedge D.C., Aparicio S.A.J.R., Behjati S., Biankin A.V., Bignell G.R., Bolli N., Borg A., Børresen-Dale A.L. (2013). Signatures of mutational processes in human cancer. Nature.

[41] Gibbs D.L. This Repo Contains the Code Necessary to Reproduce the Clusters Found in “The Immune Landscape of Cancer”. https://github.com/Gibbsdavidl/Immune-Subtype-Clustering.

[42] Pedregosa F., Varoquaux G., Gramfort A., Michel V., Thirion B., Grisel O., Blondel M., Prettenhofer P., Weiss R., Dubourg V. (2011). Scikit-learn: Machine Learning in Python. J. Mach. Learn. Res..

